# How prosocial behaviors are maintained in China: The relationship between communist authority and prosociality

**DOI:** 10.3389/fpsyg.2022.938468

**Published:** 2022-09-29

**Authors:** Jing Sheng, Shuilian Luo, Bo Jiang, Yousong Hu, Shuang Lin, Li Wang, Yashi Ren, Chunling Zhao, Zixin Liu, Jun Chen

**Affiliations:** ^1^Guangdong Key Laboratory of Mental Health and Cognitive Science, School of Psychology, Center for Studies of Psychological Application, South China Normal University, Guangzhou, China; ^2^Guangzhou Traffic School of Technology, Guangzhou Communications Technician Institute, Guangzhou, China; ^3^Teachers College, Columbia University, New York, NY, United States

**Keywords:** communist authority, secular authority, empathy, prosocial behavior, moderation, religion

## Abstract

**Objective:**

Numerous studies have demonstrated that religious belief is associated with prosocial behavior. However, how do they maintain cooperation in societies with a predominating atheist population, such as China? Different primings (explicit, subliminal, implicit) and a quasi-experiment are used to examine the link between communist authority and prosocial behaviors among college students in China.

**Materials and methods:**

In Study 1 (*N* = 398), the subjects’ communist authority in the university lab was primed by a communist-authority video. In Study 2 (*N* = 296), we compared the priming effects of communist authority and religion on prosocial intention. Study 3 (*N* = 311) investigated the priming effect of communist authority on prosocial behaviors by employing a scrambled sentence task in the university lab. A quasi-experiment was conducted in Study 4 (*N* = 313).

**Results:**

Results showed that communist-authority, a reminder of secular authorities, increased prosociality among college students. And empathy moderated the relationship between secular authorities and prosociality in Study 3 and Study 4.

**Discussion:**

Communist authority, a secular authority prime, has a positive effect on promoting prosocial behaviors. These results provided a feasible yet novel way to reveal the mechanism of the relationship between secular authorities and prosociality in China.

## Introduction

Why can humans cooperate? The effect of religion on increasing prosocial behaviors could explain cooperation and fairness among unrelated strangers in large groups and non-repeated contexts. A good deal of research has shown that individual religiosity has a vital positive influence on prosocial intentions and behaviors (Tsang et al., 2020). Specifically, priming religious conceptions subliminally or supraliminally has been found to augment cooperation (Ahmed and Hammarstedt, 2011), generosity (Shariff and Norenzayan, 2007), gratitude (Tsang et al., 2012), and charity (Pichon et al., 2007). However, the worldwide prevalence of atheists is non-trivial, numbering over half a billion or possibly more (Norenzayan and Gervais, 2013). In societies with atheism, such as China, how do they maintain cooperation? Recent work also suggests that the psychological functions of gods and governments are interchangeable (Kay et al., 2008, 2010a). In addition, the Communist Party, as the only ruling party, has a deep ruling foundation in China. The Communist authority has a strong affiliation with government authority in China. Therefore, priming communist authority in China is an effective way to prime secular authority. The necessity of investigating the effect of communist authority rather than religion on prosociality and the mechanism behind it in the Chinese context is henceforth paramount.

### Religion and prosociality

Cooperation with strangers in a one-shot and anonymous context is widely considered to be outside the extent of evolutionary mechanisms such as kin selection (Hamilton, 1964), direct (Trivers, 1971), and indirect reciprocity (Nowak and Sigmund, 2005). Those mechanisms are largely inadequate to explain the type of large-scale cooperation and normative compliance behavior found in human societies. The emergence of the idea of Big Gods (Gervais and Norenzayan, 2012b; Norenzayan, 2013; Lang et al., 2019)—powerful, morally concerned deities who are said to keep an eye on human behavior—has been proposed as one explanation for this problem. Being watched by supernatural agents has a similar effect to being watched by other people. And studies have found that even subtle cues of being watched lead people to increase prosocial behaviors (Haley and Fessler, 2005; Bateson et al., 2006). The Big Gods thus provide an effective means of maintaining social order by establishing a moral self-enforcement system powered by surveillance (Bering and Johnson, 2005; Johnson and Bering, 2006). As a result, it seems reasonable to assume that the concept of Big God evolved to allow for cooperation in human societies.

### Secular authorities and prosociality

Religious prosociality is not the world’s only source of prosocial behavior; there is evidence that secular authorities, like religion, can have a similar effect (Shariff and Norenzayan, 2007; Norenzayan, 2013). In many modern societies, secular authorities have supplanted the prosocial functions of religion. For example, Shariff and Norenzayan (2007) primed secular concepts (e.g., “civic,” “government”) to promote prosocial behaviors in the same way that religious priming does. Recent research has also expanded on the interchangeable psychological functions of gods and secular authorities, revealing that both gods and secular authorities can provide people with a sense of psychological control in the world (Kay et al., 2008, 2010b). Both watchful gods and watchful governments can encourage prosocial behavior (Gervais and Norenzayan, 2012a). When people feel their behaviors are being watched by the government at any time, they will do their best to conduct prosocial behaviors and enhance their reputation.

The significance of studying priming effects on heterogeneous samples was emphasized by Gomes and McCullough (2015) and Shariff et al. (2016). To date, most studies have been conducted in Western, Christian, or Islamic contexts. We found only two religious priming studies have been conducted in Asian countries (Clobert et al., 2015; Miyatake and Higuchi, 2017), and no prior studies on secular authority priming in Asia or with Asian samples have been published. Therefore, it’s imperative to investigate the affiliation between secular authorities and prosociality in Asia and enrich the theoretical connotation of secular prosociality.

### Communist authority—secular authorities in China

Religious beliefs promote prosocial behavior because of the supervisory role of external forces (Johnson and Bering, 2006), and for atheists, the government is the most powerful supervisory force. As mentioned above, Shariff and Norenzayan (2007) used secular concepts (e.g., “civic,” “government”) to reflect government authority. Compared to western countries, China has a different form of government. In China, the Communist Party, as the only ruling party, has a deep ruling foundation.

According to the 2013–2014 World Socialism Yellow Book released by the Chinese Academy of Social Sciences, there are about 110 million communists in the world, with 85 million of them in China. Zhong (2014) found that nearly 60% of the 7,009 urban Chinese residents polled agreed (54.9%) or strongly agreed (4.0%) that the central government would do the right thing for the people. The 2010 World Values Survey demonstrated that Chinese citizens trust political institutions more than the global average (Yang and Tang, 2010). According to the 2018 World Values Survey, 95% of Chinese citizens have considerable trust in the national government. Furthermore, in the face of a coronavirus pandemic, a survey of nearly 20,000 people from 31 provinces across China revealed that Chinese citizens’ trust in their national government increased to 98 percent (Wu, 2021). To sum up, the Communist Party of China (CPC) has enough credibility and authority over the Chinese people. At the Sixth Plenary Session of the 18th CPC Central Committee, it was proposed that the “lofty ideal of communism and the common ideal of socialism with Chinese characteristics” is the spiritual pillar and politics of Chinese Communists and should be fostered. The CPC’s primary goal is to serve the people wholeheartedly (Kai, 2017), and its original intention and mission is to work for the happiness of the Chinese people and the rejuvenation of the Chinese nation. Different regimes and values in various countries may influence the prime of individual secular authority in various regions. Because of China’s unique form of the regime and its communist values, communist authority in China has a stronger affiliation with prosociality than secular authority (Yang and Wiepking, 2021). In order to improve the ecological validity of this research, the communist-authority prime, as a special secular prime, was applied.

### Empathy as a moderator

Empathy is an emotional regulation process used to alleviate personal suffering caused by the pain or discomfort of others, allowing compassionate and helpful behavior toward others to be mobilized (Decety and Lamm, 2009). Thus, the awareness of others’ states, thoughts, and feelings is essential for helping behavior (Coke et al., 1978). Since empathic concern arises from the blurring of the boundaries between the self and the other, the underlying motivation of the link between empathy and prosocial behaviors is that empathy transforms prosocial behaviors into acts toward oneself (Cialdini et al., 1997). For example, Van Lange (2008) induced empathic concern by having participants read a piece of pathetic information coming from another imaginary research participant. Results showed that higher levels of empathic concern for another participant were associated with more subsequent prosocial choices. Consistent with prior work, empathic concern also moderated the effects of opponents’ strategy on cooperation (Rumble et al., 2010).

Previous researchers have investigated the relationship between religion and empathy (Haneef Khan et al., 2005). Empathy or mentalizing abilities (the ability to perceive and attribute mind to other beings) appear to be vital for religious beliefs (Norenzayan et al., 2012; Gervais, 2013b). For example, Markstrom et al. (2010) suggested a positive connection between empathy and the importance of religious beliefs. The capability to attribute the mind to another being (human or supernatural) is thought to be a necessary condition for developing religious beliefs, as deities are frequently regarded to be intentional agents with their mental states (Gervais, 2013b). As mentioned above, the watchful agents, the gods, and the government authority, both promote prosocial behavior. Further, it can be inferred that individuals with high empathy can better perceive the supervision of the government authority and have more prosocial behaviors.

### The present research

This research aims to investigate whether communist authority could increase the prosociality of participants in the Asian context and whether empathy could moderate the relationship between communist authority and prosociality. The role of priming communist authority in activating prosociality was tested across four experiments. In Study 1, we examine the effect of explicit communist authority on prosocial intentions among college students. In Study 2, we compared the priming effects of communist authority against the religious prime. Buddhism promoted the values of love, peace, harmony, and tolerance (Goodman, 2014; Prakash, 2018). Guan et al. (2018) also proved that priming Buddhist concepts explicitly or implicitly increased participants’ prosociality in China. Therefore, in Study 2, Buddhism prime, an effective religious prime in China, was applied. The participants’ prosocial intentions were measured using a series of hypothetical scenarios in Study 1 and Study 2. In Study 3, the impact of implicit communist authority on the actual prosocial behaviors was tested using an economic game called the dictator game. A quasi-experiment was conducted in Study 4. The amount of charitable giving is used as an indicator of pro-social behavior. Participants in four experiments came from different universities in three cities in south China.

Both secular authority and religious beliefs can exert a strong supervisory influence on individual behavior, we propose that communist authority can increase prosocial behaviors and empathy also plays a key role in communist authority and prosocial behaviors: H1. Priming the communist-authority video would significantly increase subjects’ prosocial intentions more than the neutral-prime group and the no-prime group; H2. Participants who had been primed with Buddhism or communism would have a similar effect on prosocial intentions; H3. Compared to the control group, subjects who were primed with communist-authority words in a scrambled sentence task would conduct more prosocial behaviors. In the prime group, participants with high empathy will show more prosocial behaviors than participants with low empathy; H4. Participants will present more prosocial behaviors after listening to a communist-authority lecture than participants listening to a physic lecture instead; empathy moderates the link between communist authority and prosociality.

## Study 1

In Study 1, we compared differences in prosocial intentions across three conditions to investigate if communist authority increases prosocial intentions more.

### Method

#### Participants

A target sample size of 252 was determined based on an *a priori* power analysis (Faul et al., 2007) to ensure sufficient power (i.e., power > 0.95) to detect a medium between-subjects effect (*f* = 0.25) with an alpha criterion (*p* < 0.05) in a two-tailed one-way ANOVA. Thus, 450 subjects were recruited through posters displayed at the university in south China and randomly assigned to either the communist-authority-prime, neutral-prime, or no-prime condition. 52 participants were excluded from the analysis due to missing data or watching videos carelessly (15 subjects in the communist-authority-prime group; 23 subjects in the neutral-prime group; 14 subjects in the neutral-prime group). The standard of watching video carelessly was based on the score of the recognition test. So, there were 398 subjects left in the study. 135 subjects in the communist-authority-prime group (*M*_*age*_ = 19.56 years; *SD* = 1.01), 127 subjects in the neural-prime group (*M*_*age*_ = 18.84 years; *SD* = 1.23) and 136 subjects in the no-prime group (*M_*age*_* = 18.98 years; *SD* = 1.23). It should be noted that all the participants in the four studies are not majoring in Marxism. The three groups were balanced on sex, volunteering, and political identity, and the details are presented in Supplementary Table 1. Each subject was paid 15 CNY for this experiment. Informed consent for four studies was obtained from all subjects. All four procedures of the four studies were approved by the ethics of the human research committee of the author’s university.

#### Materials

##### Priming videos

The video materials contain a communist-authority video and a neutral-prime video. The communist-authority video was excerpted from the video of the 19th National Congress of the Communist Party of China and lasted 3 min; and the neutral-prime video was a popular science video that lasted 3 min, introducing the physical phenomena and principles of light refraction. The communist-authority video focuses on the development of the Communist Party of China and its historical achievements. Then 23 graduate students in psychology were asked to assess the arousal and connotation of two videos on a 7-point Likert scale ranging from 1 to 7 (1 = calm and 7 = excited, for arousal; 1 = not related to communism, government authority, and 7 = related to communism, government authority; for connotation). The mean score of arousal for video 1 (*M* = 2.52, *SD* = 1.08) and video 2 (*M* = 2.26, *SD* = 1.39, *t* = 0.70, *p* = 0.49, *M*_*diff*_ [95% CI] = 0.26 [-0.51, 1.03]) showed no difference. The mean score of the two items connotation for neutral-prime video (*M* = 2.87, *SD* = 1.06) was significantly below the communist-authority video (*M* = 5.26, *SD* = 1.39), indicating the two videos met the requirements of the experiment (*t* = 6.56, *p* < 0.001, *M*_*diff*_ [95%CI] = 5.40 [4.95, 5.84]).

##### No-prime group

There is no video-prime in the no-prime group, and subjects just need to finish prosocial intention measure. For more details, see Supplementary Figure 1.

##### Communist belief scale

Communist Belief Scale (McFarland, 1998) (Cronbach’s α = 0.78) has 28 items and was designed to utilize a 5-point Likert scale, with 1 representing strong disagreement, 5 representing strong agreement. The higher the score is, the stronger the communist belief of the students is.

##### Prosocial tendencies measure

Prosocial Tendencies Measure (PTM) (Carlo and Randall, 2002) has 23 items, such as “I tend to help people who are in a real crisis or need.” Participants were asked to rate the extent to which statements described themselves on a 5-point scale ranging from 1 (strongly disagree) to 5 (strongly agree). After calculating the total score of PTM, the higher the score is, the stronger the prosocial intentions are (Cronbach’s α = 0.85).

##### Duke university religion index

The Duke University Religion Index (DUREL) (Koenig and Arndt, 2010) is a widely used scale for assessing religiosity. It is a 5-item scale that measures organizational religious events, non-organizational religious events, and intrinsic religiosity. The Chinese version of this scale was utilized in the study, and it has good reliability and validity (Hanhui et al., 2014; Cronbach’s α = 0.74).

##### Prosocial intentions task

The participants’ prosocial intentions were measured using a series of hypothetical scenarios adapted from prior studies (Nelson and Norton, 2005; Jordan et al., 2011). Similar hypothetical scenario paradigms have also been used in multiple studies to measure prosocial intentions or tendencies. Then the participants were asked to rate to what extent they would like to offer help on a 9-point scale ranging from 1 (not willing) to 9 (strongly willing). A prosocial intention score was computed for each participant by taking the mean of the response to each of the four prosocial activity questions.

##### Recognition test

The recognition test was used to check whether the subjects had watched the video earnestly. There were twenty-one words in the recognition test and six of them were mentioned in the communist-authority video; six of them were mentioned in the neutral video; and nine of them were unrelated to either of the two videos. Unrelated words include fence, bamboo, hand saw, and so on. After watching the video, participants were required to mark the six words that appeared in the video they just watched. The parts of speech and the number of words were matched among all the words. The right answer scores one point. A total score, ranging from 0 to 6, above 3, means that subjects were included in the final statistics.

##### Manipulation check

After the recognition test, participants were asked to rate one manipulation check item on a 9-point scale ranging from 1 (strongly disagree) to 9 (strongly agree) as follows: I believe in the principles of the Communist Party.

All measures, data, and analysis code are available at the Open Science Framework (OSF).^1^

#### Procedure

All subjects were seated in private and quiet rooms watching the video during the experiment. Then participants in the communist-authority-prime and neutral-prime groups were required to finish the recognition test, followed by the manipulation check, the prosocial intention task, some control variables (CBS, PTM, DUREL) and demographic information. Subjects in the no-prime group were required to complete the manipulation check, followed by the prosocial intention task, and demographic information.

### Results and discussion

#### Manipulation check

Using one-way ANOVA, the overall effect of the manipulation was significant, *F* (2,395) = 43.83, *p* < 0.001, η*_*p*_^2^* = 0.18. All multiple comparisons were conducted with Bonferroni correction in Study 1. Specifically, the effect of the manipulation in communist-prime group (*M* = 6.02, *SD* = 1.43, 95% CI [5.78, 6.27]) was significantly higher than that in the neutral-prime group (*M* = 4.50, *SD* = 1.54, 95% CI [4.23, 4.77], *p* < 0.001) and no-prime group (*M* = 4.52, *SD* = 1.62, 95% CI [4.24, 4.79], *p* < 0.001), the difference of manipulation between neutral-prime group and no-prime group was not significant, indicating the communist authority was successfully primed.

#### Scores of the prosocial intentions

Demographic information and control variables (CBS, PTM, DURI) in each group are presented in Supplementary Table 1, which means the communist belief, prosocial tendency, religion, and demographic information of participants were balanced among the three groups.

**FIGURE 1:**
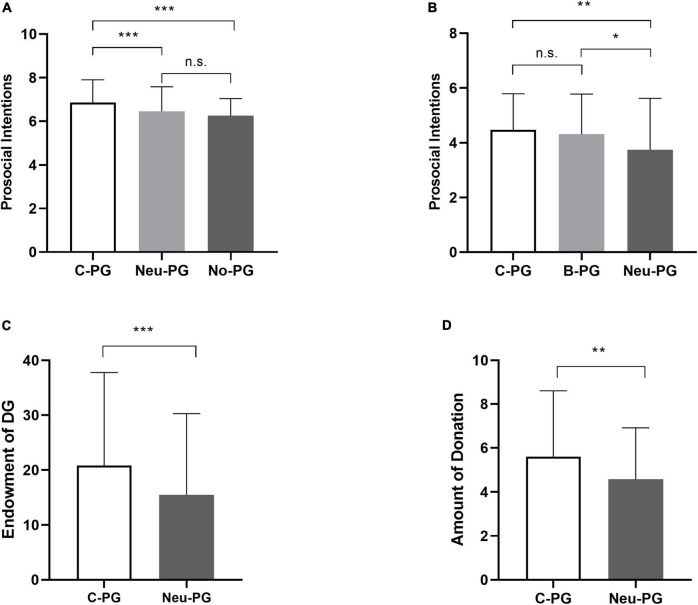
**(A)** The difference in prosocial intentions among three groups after video-prime. **(B)** The difference in prosocial intentions after the scrambled sentence task between three groups. **(C)** The endowment of DG after the scrambled sentence task in the prime and control groups. **(D)** The difference in donation rates between the communist-authority and control groups. Ca-PG means communist-authority-prime group; B-PG means Buddhist-prime group; Neu-PG means neutral-prime group; No-PG means no-prime group; DG means dictator game. ***Means *p* < 0.001; ** means *p* < 0.01; * means *p* < 0.05; n.s., none significant.

We applied the Welch F test to analyze participants’ scores on the prosocial intentions task when the variances of the dependent variable were non-homogenous across groups (Delacre et al., 2019). The results showed that improvement in the prosocial intentions in the communist-authority-prime group was significantly greater than in the neutral-prime group and no-prime group [*F* (2, 253.23) = 14.22, *p* < 0.001, *η_*p*_^2^* = 0.06]. The subjects’ prosocial intentions in the communist-authority-prime group (*M* = 6.86, *SD* = 1.05, 95% CI [6.68, 7.03]) was significantly higher than the neutral-prime group (*M* = 6.46, *SD* = 1.13, 95% CI [6.26, 6.66], *p* = 0.004) and the no-prime group (*M* = 6.26, *SD* = 0.78, 95% CI [6.12, 6.39], *p* < 0.001). The difference in prosocial behavior intention between the neutral-prime group and the no-prime group was not significant (*p* = 0.29, *M*_*diff*_ [95% CI] = 0.21 [-0.09, 0.50]; Figure 1A).

To our knowledge, this is the first study investigating the effect of communist authority on prosocial intentions in China. In this study, communist authority was successfully activated in the communist-authority group after watching the communist-authority video. They would express more prosocial intentions relative to the neutral-prime group and no-prime group. Meanwhile, the mean score of the neutral-prime group was a little bit higher than the no-prime group, but not remarkable in statistics, further explaining why the priming of the popular science video was neutral. The results of Study 1 only proved that communist authority increased individuals’ prosocial behavior. But it’s not sure that communist authority in promoting prosocial behaviors serves as an embodiment of secular authority, as the introduction speculates. And whether secular authority and religion play similar roles in promoting pro-social behavior in China.

## Study 2

In Study 2, we sought to replicate and expand the findings of the first study. We introduced an additional priming condition to examine the strength of prosocial intentions of the communist-authority relative to religion. And before the experiment, we investigated whether communist authority was a manifestation of secular authority.

### Method

#### Participants

A target sample size of 252 was determined based on an *a priori* power analysis (Faul et al., 2007) to ensure sufficient power (i.e., power > 0.95) to detect a medium between-subjects effect (*f* = 0.25) with an alpha criterion (*p* < 0.05) in a two-tailed one-way ANOVA. Thus, 300 subjects were recruited from another university in a city in south China and randomly assigned to either the communist-authority-prime, Buddhist-prime, or neural-prime condition. 1 subject in the communist-authority-prime group and 3 subjects in the Buddhist-prime group were excluded because their responses to the funneled debriefing questions suggested awareness of the purpose of the present study. Thus, a total of 296 participants provided data for the analysis (none of the participants were majoring in Marxism). 99 subjects in the communist-authority-prime group (*M*_*age*_ = 20.54 years; *SD* = 1.22), 97 subjects in the Buddhist-prime group (*M*_*age*_ = 19.63 years; *SD* = 1.02), 100 subjects in the neural-prime group (*M*_*age*_ = 20.06 years; *SD* = 1.26). Each subject was paid 15 CNY for this experiment.

#### Materials

##### Pre-test

In order to demonstrate the effectiveness of the communist-authority words in priming secular authority and the interchangeable function between communist authority and religion, we conducted a pre-test for experimental materials.

Thirty participants evaluated forty Buddhist words, forty communist-authority words, and forty neutral words. Both arousal and connotation were assessed through a 7-points scale (for arousal, 1 = calm and 7 = excited; for connotation, 1 = not related to Buddhism/communism and 7 = related to Buddhism/communism). While only the corresponding attributes of two sets of priming words must be evaluated, both attributes of neutral words must be evaluated. Finally, 20 communist-authority words (e.g., “red army”), 20 Buddhist words (e.g., “Buddha”), and 20 neutral words (e.g., “shirt”) were chosen for the current study (more details can be seen in the Supplementary material of Study 2). The Buddhist, communist-authority and neutral priming words differed significantly in terms of connotation [neutral: *M* = 1.65, *SD* = 0.38; Buddhist: *M* = 5.77, *SD* = 1.01; communist-authority: *M* = 5.60,*SD* = 1.00; neutral vs. Buddhist: *t* (29) = 19.61, *p* < 0.001; neutral vs. communist-authority: *t* (29) = 19.51, *p* < 0.001], whereas the Buddhist, communist-authority and neutral priming words showed no significant difference in terms of arousal [neutral: *M* = 2.433, *SD* = 1.45; Buddhist: *M* = 2.67, *SD* = 1.27; communist-authority: *M* = 2.83, *SD* = 0.95; neutral vs. Buddhist: *t* (29) = 0.59, *p* = 0.56; neutral vs. communist-authority: *t* (29) = 1.45, *p* = 0.16].

Then the connotation of communist-authority words and secular authority words were also rated. These secular authority words were adapted from Shariff and Norenzayan (2007) early research. The item is as follows: How relevant do you think this word is to government authority (1 = not related to government authority and 7 = related to government authority)? Communist-authority words and secular authority words showed a marginal significant difference in terms of connotation [communist-authority words: *M* = 5.80, *SD* = 1.00; secular authority words: *M* = 5.30, *SD* = 1.02; *t* (58) = 1.92, *p* = 0.06]. In terms of data results, communist-authority words can represent government authority similarly or even better than secular authority words, which supports our hypothesis that priming communist authority in China is more effective prime of government authority.

##### Lexical decision task

Participants were required to make decisions about a series of letter strings displayed on a screen. At each trial, a string of Xs appeared followed by some Chinese characters. Participants needed to decide whether the Chinese characters were actual words or not. If the Chinese characters were a word, participants were informed to press the “Yes” key, and if not, they had to press the “No” key. The whole task consists of 20 lexical decision trials.

The LDT was adapted from Wittenbrink et al. (1997, 2001). The experiment was programmed using E-Prime 2.0 software (Psychology Software Tools, Inc., Pittsburgh, PA, United States^2^). On each trial, a fixation point appeared for 500ms on the screen. Then a prime appeared for 15 ms before being covered by a mask. After 500 ms, the mask was replaced by Chinese characters, which vanished after 500 ms. Then the computer stopped until participants responded by pressing the corresponding key (“Yes” or “No”). The Chinese characters were made up of 20 words, specifically 10 neutral words and 10 non-words. In different conditions, each letter string was followed by a word (e.g., Buddha) related to Buddhism in the religious condition, a word (e.g., Marx) related to communism in the secular condition, and a neutral word (e.g., shirt) in the neutral condition.

##### Repeated variables

As in Study 1, participants were given the CBS (Cronbach’s α = 0.73), PTM (Cronbach’s α = 0.86), DUREL (Cronbach’s α = 0.68), PIT (Cronbach’s α = 0.63) and the same demographic questions as in Study 1.

#### Procedure

First, participants needed to finish LDT, then the prosocial intention task, some scales, and demographic information. We wanted to covertly prime our participants. Therefore, subjects needed to answer two check questions to ensure that participants were not aware of the relationship between the priming manipulation and the subsequent experimental task (the prosocial intention task). The questions are as follows: (1) Do you think there are any themes and rules in sentences during the language task? (2) Do you think the tasks are related to each other? After the study, all participants got 15 CNY for this experiment.

### Results and discussion

Demographic information and control variables in each group are presented in Supplementary Table 1. A one-way ANOVA (Prime Type: communist-authority-prime group vs. Buddhist-prime group vs. neutral group) was used. Participants primed with Buddhist words (*M* = 4.31, *SD* = 1.47, 95% CI [4.01, 4.60], *p* = 0.03, *M*_*diff*_ [95% CI] = 0.57 [0.03, 1.11]) and communist-authority words (*M* = 4.47, *SD* = 1.31, 95% CI[4.21, 4.73], *p* = 0.004, *M*_*diff*_ [95% CI] = 0.73 [0.19, 1.27]) showed more prosocial intentions than participants primed with neutral words {*M* = 3.74, *SD* = 1.89, 95% CI[3.36, 4.11], *F* (2, 296) = 5.92, *p* = 0.003, *η_*p*_^2^* = 0.03}. The difference in prosocial intentions between the communist-authority-prime group and the Buddhist-prime group was not significant (*p* = 0.95, *M*_*diff*_ [95% CI] = 0.16 [-0.38, 0.70]; Figure 1B).

This study suggests that the communist-authority prime in China is an effective way to prime government authority. Furthermore, given that neutral words were used in the control condition, and the suspicion probe revealed little reflective awareness of the communist and Buddhist nature of the prime, we can rule out the possibility that the effect of communist-authority and Buddhist concepts on prosocial intention was an artifact of the priming procedure itself or was a by-product of demand characteristics. Finally, and most importantly, we showed that subliminal activation of concepts related to communist authority restrained selfishness as much as religious suggestion did in China. Thus, this study further demonstrated the effectiveness of the communist-authority prime in promoting prosocial intention and further hints at the similarity in the psychological functions between secular authority (specifically, communist authority) and religion.

## Study 3

In Study 3, the role of priming communist authority on prosocial behaviors was further investigated through an implicit prime. There were several extensions compared with Study 2: (a) Participants were primed implicitly by the scrambled sentence task; (b) actual prosocial behavior was measured by an economic game; (c) empathy was tested as a moderator.

### Method

#### Participants

A target sample size of 328 was determined based on an *a priori* power analysis (Faul et al., 2007) to ensure sufficient power (i.e., power > 0.95) to detect a between-subjects effect (*d* = 0.40) with an alpha criterion (*p* < 0.05) in a two-tailed between-subjects *t*-test. In fact, a total of 170 (communist-authority-prime group) and 170 (neutral-prime group) volunteers were recruited from another university in another city in south China. All participants were randomly assigned to either the communist-authority prime or the neutral-prime condition. There were 15 (communist-authority-prime group) and 14 (neutral-prime group) participants who were not included in subsequent analyses due to missing data. At last, 155 (communist-authority-prime group, 75 female, *M*_*age*_ ± *SD* = 20.47 ± 1.30) and 156 (neutral-prime group, 83 female, *M*_*age*_ ± *SD* = 19.69 ± 1.03) participants were included in the final statistics. A sensitivity analysis of the current sample size was calculated with G*Power 3.1.9.2 (Faul et al., 2007) (*d* = 0.41).

#### Materials

##### The scrambled sentence task

We used the scrambled sentence task of Srull and Wyer (1979) to prime our subjects. Half of the participants received a prime with communist authority representations and half of the participants received a prime with neutral representations. All participants were required to construct coherent and grammatically correct five-word sentences out of 20 sets of six words by eliminating one of the words. All the words were presented in random order. Those in the neutral condition were given words with no communist-authority connotation. For those in the communist-authority prime condition, ten of the 20 scrambled sentences contained words that were associated with communist authority and ten that were not. All materials are presented in Chinese. For example, if the participants received the scrambled sentence “faster than sound direction travels light,” they were expected to write “light travels faster than sound” by eliminating “direction” (see the Supplementary material of Study 3 for specific details).

##### The dictator game

The dictator game, introduced by Kahneman et al. (1986), is a one-shot/two-person game in which the dictator must decide how to distribute a sum of money between herself or himself while the recipient must accept the dictator’s decision. Participants were given the following instructions:

*You have been given 100 tokens (1 token* = *0.2 CNY* = *0.031 USD) in this situation. You can send some, all, or nothing to another person that you have randomly been paired with. The other person has not received any money. The money you will have after your decision is 100 tokens minus the amount you send to the other person.*

*The other person will only have the amount you send. Please state the amount of money (0-100 tokens) you would like to send to the other person.*

##### Interpersonal reactivity index

The Interpersonal Reactivity Index (Davis, 1980) has 28 items and contains two subscales: empathic concern, which taps affective aspects of empathy; and perspective-taking, which taps cognitive aspects. Participants scored the items on a 5-point scale, ranging from 0 (doesn’t describe me at all) to 4 (describes me very well). The Interpersonal Reactivity Index has adequate reliability and validity (Cronbach’s α = 0.89).

##### Repeated variables

Communist belief scale (CBS) (Cronbach’s α = 0.67), PTM (Cronbach’s α = 0.89), DUREL (Cronbach’s α = 0.80) and the same demographic questions as in Study 1 were administered to participants.

#### Procedure

The experiment was a paper-pencil experiment. Participants were told that this study was to assess the comprehensive abilities of college students, including three parts: a language proficiency task, an economic decision-making ability task, and an attitudes survey. Once participants were seated, they received their show-up fee of 20 CNY (20 CNY = U.S. $3.11) and a booklet with the scrambled sentence task on the first page that they were instructed to do first. Then they were required to turn the page and make a decision on the dictator game. After the booklet containing the scrambled sentence task and the experimental games had been collected, participants responded to some questionnaires. Finally, two funneled debriefing questions (as in Study 2) were used to probe for suspicion. After the study, all participants got their final tokens converted into real money. For each token left, we paid 0.2 CNY. A hundred tokens is the equivalent of over $3, and can reasonably be used to pay for a 15-min taxi ride, several kilos of fruit, or lunch in China. All participants completed the task in a separate room, and the task lasted about 15 min.

### Results and discussion

#### The effect of communist-authority priming on the dictator game

Demographic information and control variables in each group are presented in Supplementary Table 2. No subject was aware of the relationship between communist-authority prime manipulation and the experimental task. Using independent-samples *t*-tests, in the dictator game, the donated amount in the communist-authority-prime group (*M* = 20.85, *SD* = 16.93) was higher than in the neutral-prime group {*M* = 15.50, *SD* = 14.76, *t* (309) = 2.97, *p* = 0.003, *d* = 0.34, *M*_*diff*_ [95% CI] = 5.35 [1.81, 8.90]} (Figure 1C), indicating that participants in the communist-authority-prime group would show more generous behavior than in the neutral-prime group.

#### Empathy as a moderator

Model 1 of the PROCESS macro (Hayes, 2013) was used to conduct a moderated regression analysis. Analyses illustrated that participants’ high empathy predicted greater prosocial behavior. And we found that the predicted interaction between group and empathy was significant, *b* = 0.26, *SE* = 0.16, *t* = 2.28, *p* = 0.03, 95% CI [0.04, 0.49]. A simple slope analysis revealed that higher empathy was associated with a stronger positive relationship between the group and the endowment of DG (one SD above the mean), *b* = 0.88, *t* = 5.86, *p* < 0.001, 95% CI [0.58, 1.17]. When empathy was lower, the relationship remained positive, but the strength of the effect was significantly weaker, *b* = 0.35, *t* = 2.11, *p* = 0.04, 95% CI [0.02, 0.68] (Supplementary Figure 2A).

An implicit priming effect of communist authority on true prosocial behavior was implied in Study 3 to eliminate participants’ demand characteristics. Results of Study 3 were in line with the previous two studies, which further illustrated that the communist-authority prime, a prime of secular authorities, can significantly enhance actual cooperation in an economic game. And empathy significantly moderated the link between communist authority and prosocial behavior. In other words, participants with higher empathy in both groups were more likely to donate money than participants with lower empathy.

## Study 4

In order to improve the robustness and ecological validity of experimental research, we used a more realistic priming method in Study 4.

### Method

#### Participants

A target sample size of 328 was determined based on sensitivity analysis (Faul et al., 2007) to ensure sufficient power (i.e., power > 0.95) to detect a between-subjects effect (*d* = 0.40) with an alpha criterion (*p* < 0.05) in a two-tailed between-subjects *t*-test. In fact, a total of 154 (communist-lecture group, 111 female, *M_*age*_* ± *SD* = 20.76 ± 0.83) and 146 (physics-lecture group, 94 female, *M_*age*_* ± *SD* = 20.01 ± 0.77) volunteers who donated money to Half the Sky Foundation were recruited from another university in another city in south China. Participants were recruited from the two lectures directly, and there was no experimental manipulation in this study.

#### Materials

##### Communist lecture

This lecture was attended by almost two hundred students, lasted 1 h and 20 min, and was mainly about three parts: (1) What is Marxism; (2) Why should one become a Marxist; (3) How to be a staunch Marxist.

##### Physics lecture

Approximately two hundred and forty students attended the physics class. This lecture lasted for 1 h and 20 min s and was mainly about four parts: (1) What is physics; (2) Physical phenomena in life; (3) The principles of Physics behind phenomena; (4) Frontiers of research in Physics.

All subjects were students who came to the two lectures voluntarily and were unaware that there would be a charitable donation at the end of the lecture.

##### Charitable appeal

There was a brief introduction of a fictitious non-profit organization called Half the Sky Foundation in the charitable appeal. The phrase “half the sky” is, in the Chinese language, intimately linked with the concepts of interdependence, helping, and self-sacrifice. It is derived from the Chinese proverb “Women hold up half the sky.” The description of the organization explained that the charity’s mission was to assist with the educational and developmental needs of children in the remote and rural Guangdong province of China.

#### Repeated variables

Participants were also given the CBS (Cronbach’s α = 0.70), PTM (Cronbach’s α = 0. 86), DUREL (Cronbach’s α = 0.67), IRI (Cronbach’s α = 0.85) and the same demographic questions as in Study 1.

#### Procedure

The experiment was conducted at another university in Guangdong on August 5, 2021. At the end of the two lectures, the experimenter entered the room and showed participants a printed charity appeal for the Half the Sky Foundation. Meanwhile, a collection box had been placed near the exit of the classroom to assess charitable giving. The amount of money (1 CNY = 0.16 USD) that participants placed in the box served as an index of charitable giving. Participants were contacted after the study and were asked to finish an online survey via Sojump (an online crowdsourcing platform in mainland China that provides functions equivalent to Amazon’s Mechanical Turk^3^). Sojump has 2.6 million nationwide registered members. There have been many previous works that used Sojump to collect experimental data published (He et al., 2017; She et al., 2017; Wang et al., 2019). After the experiment, the money they had donated was returned to them *via* WeChat account transfer. At last, we assigned subjects who donated money after physics class to the physics-lecture-prime group, and subjects who donated money after communist-authority class to the communist-authority-prime group in statistics.

### Results and discussion

#### The effect of the communist lecture on donation

Demographic information and control variables in each group are presented in Supplementary Table 2. Using independent-samples *t*-tests, the amount of donation in the communist-lecture group (*M* = 5.60, *SD* = 3.00) was significantly higher than in the physics-lecture group {*M* = 4.58, *SD* = 2.33, *t* (298) = 3.28, *p* = 0.001, *d* = 0.38, *M*_*diff*_ [95% CI] = 1.02 [0.41, 1.63]}, indicating that participants in the communist-lecture group would show more prosocial behavior than the physics-lecture group (Figure 1D).

#### Empathy as a moderator

Model 1 of the PROCESS macro (Hayes, 2013) was used to conduct a moderated regression analysis. In charitable giving, analyses illustrated that participants’ high empathy predicted greater prosocial behavior.

And we found that the predicted interaction between group and empathy was significant, *b* = 0.26, *SE* = 0.11, *t* = 2.32, *p* = 0.02, 95% CI [0.04, 0.49]. A simple slope analysis revealed that higher empathy was associated with a stronger positive relationship between the group and the amount of donation (one SD above the mean), *b* = 0.61, *t* = 3.87, *p* = 0.0001, 95% CI [0.30, 0.92]. When empathy was lower, the relationship remained positive, but the strength of the effect was significantly weaker (one SD below the mean), *b* = 0.08, *t* = 0.52, *p* = 0.60, 95% CI [-0.23, 0.40] (Supplementary Figure 2B).

A quasi-experiment cannot reveal the causal effect of communist authority on prosocial behavior. According to the results of Study 4, we could cautiously infer that there was a strong connection between communist authority and prosociality. Similarly, the interaction between group and empathy influences charitable donation: in the communist-lecture condition, high-empathy participants showed more prosocial behaviors than low-empathy participants.

## General discussion

In this research, we examined the influence of secular authorities on prosociality. In Study 1, by using explicit priming (a communist-authority video), we showed that the communist-authority prime had the power to elicit prosocial intentions. Study 2 revealed the feasibility of using communist authority to denote secular authority in China. In addition, it further revealed a similar effect of communist-authority prime and religious prime on prosocial intentions using a subliminal prime task (a lexical decision task). Study 3 showed that implicitly priming communist authority promoted actual prosocial behavior. And the relationship between communist-authority prime and prosociality was moderated by empathy. Shariff and Norenzayan (2007) primed secular concepts to promote prosocial behaviors in the same way that religious priming does. These results support Shariff and Norenzayan (2007) findings that priming communist-authority concepts promotes prosocial behaviors just as effectively as religious representations, and empathy is positively correlated with secular authorities and prosociality (Van Lange, 2008; Markstrom et al., 2010; Hardy et al., 2012). In addition, empathy moderates the effect of the communist-authority prime on prosocial behaviors (Rumble et al., 2010). There are concerns regarding the generalizability and ecological validity of experimental research since most studies have taken place in laboratory settings and/or utilized artificial stimuli. Thus, we performed a quasi-experiment in Study 4 to address these issues with a result that still replicated the results of the previous three studies.

Previous findings have demonstrated that gods and governments can serve similar psychological and social functions (Gervais and Norenzayan, 2012b). More precisely, both religious and secular authorities are conducive to relieving people’s existential concerns and giving people a sense of supervision in the unpredictable world (Kay et al., 2008). Human are hypersensitive to cues that others are watching. When people feel their actions are being watched, they will do their best to enhance their reputation (Haley and Fessler, 2005; Bateson et al., 2006). Being watched by God or the government for humans at any given time can then increase both public self-awareness and prosociality (Gervais and Norenzayan, 2012a). Thus, people living in places with more powerful governments, such as China, had a smaller religious population but a higher number of prosocial behaviors. The CPC has enough credibility and authority with the Chinese people, and communist authority has a strong affiliation with government authority in China. Therefore, priming communist authority can make subjects feel like they are being watched by the government, leading to more prosocial behavior.

In addition, there was another explanation for the communist authority’s influence on pro-social behavior. Different countries have different types of regimes and values. In western countries, such as the United States, where the government is associated with capitalist values. In China, the government is associated with communist values. The CPC’s primary goal is to serve the people wholeheartedly (Kai, 2017) and its original intention and mission are to work for the happiness of the Chinese people and the rejuvenation of the Chinese nation. As a result, the values that underpin communist authority may predispose people to more pro-social behavior. The current study, however, does not fully distinguish between the supervisory role of government authority and communist values. Furthermore, the goal of our research is to determine whether communist authority encourages pro-social behavior. To summarize, the prime of communist authority can promote pro-social behavior, either because of the government’s supervisory role or because of the prosociality of communist values.

Our findings also demonstrated that empathy acts as a moderator and works on the relationship between communist-authority prime and prosociality. Compared to people with lower empathy, priming with communist authority could significantly predict more cooperation and generosity when people had relatively strong empathy. This finding is not only consistent with previous studies (Markstrom et al., 2010; Hardy et al., 2012) but also reveals the mechanism of communist authority as it pertains to prosocial behaviors. It is widely suggested that high levels of empathy or mentalizing abilities are essential to developing religious beliefs as people usually think of deities as intentional agents with their own mental states (Gervais, 2013b). Since empathy stems from blurring the boundaries between the self and the other, prosocial behaviors are transformed into acts toward oneself (Cialdini et al., 1997). In other words, people with high empathy are inclined to behave prosocially. Secular authority plays a psychological function similar to religion in promoting prosocial behavior with more empathy. Thus, participants with higher empathy in the communist-authority group were more likely to donate money than participants with lower empathy.

This study is not without limitations: the sample of this research is normal college students in China. In the future, these results should be replicated in samples of different ages and different countries. Second, participants’ communist beliefs were measured after the task rather than before it. And in Study 4, students who volunteered for the communist lecture might have more prosocial tendencies than those who volunteered for the physics lecture. But the scores of PTM between the two groups were not significant, which somehow illustrates the role of communist authority in promoting pro-social behavior. Last, the current study fails to distinguish fully between the supervisory role of governmental authority and communist values. In the future, this cultural specificity should be taken into account when comparing the results of similar experiments across countries.

Why can humans, even atheists, cooperate? Our studies may offer an explanation: communist authority promotes prosocial intentions and behaviors. This is the first study in Asia (in particular, this is a study done in China, one of the few collectivist countries) that identified the link between communist authority and prosocial behaviors. Our findings extend the implications of previous research by secular authorities on prosociality and provide possible viewpoints to further understand prosocial behaviors.

## Data availability statement

The datasets presented in this study can be found in online repositories. The names of the repository/repositories and accession number(s) can be found below: https://osf.io/kam62/?view_only=73e2bf6b3d0947878a0fddc59ec646fa.

## Ethics statement

The studies involving human participants were reviewed and approved by the Ethics in the Human Research Committee of the South China Normal University. The patients/participants provided their written informed consent to participate in this study.

## Author contributions

JS, BJ, and JC designed the research. JS, BJ, YR, and CZ performed the experiments. JS, YR, CZ, and ZL analyzed the data. JS and SLi interpreted results of the experiments. JS wrote the main manuscript text and prepared the figures. JS, SLu, LW, YH, and ZL edited and revised the manuscript. All authors reviewed and approved the manuscript.

## Footnotes

1
https://osf.io/kam62/

2
www.pstnet.com/eprime

3
https://www.wjx.cn/
