# Supplementary material for: How prosocial behaviors are maintained in China: The relationship between communist authority and prosociality
Source: Front Psychol. 2022 Sep 29;13:938468. doi: 10.3389/fpsyg.2022.938468 (PMC9558272; doi:10.3389/fpsyg.2022.938468)
Supplement: Supplementary file 1 [file Data_Sheet_1.pdf]

Supplementary Table 1 The descriptive results of demographic questions and control variables of two studies.

	**Study 1** (n = 398)			P_1	**Study 2** (n = 296)			P_2
	C-PG (n = 135)	Neu-PG (n = 127)	No-PG (n = 136)		C-PG (n = 99)	B-PG (n = 97)	Neu-PG (n = 100)	
Sex (male/female)	48/87	43/84	53/83	0.68[a]	51/48	45/52	56/44	0.40[a]
Volunteering (yes/no)	66/69	71/56	64/72	0.32[a]	89/10	92/5	88/12	0.23[a]
Political identity (communist/nonparty)	69/66	75/52	75/61	0.43[a]	9/90	10/87	10/90	0.96[a]
CBS	92.04±9.67	90.41±9.93	91.78±9.66	0.35	98.08±13.85	99.14±10.02	100.12±9.08	0.44
PTM	83.07±10.10	81.77±10.39	82.75±10.51	0.58	82.13±12.39	81.04±10.31	80.29±11.97	0.53
DUREL	7.17±2.42	7.36±2.04	7.33±2.33	0.76	7.09±2.37	7.34±2.32	7.52±1.06	0.32

Note. CBS, Communist Belief Scale; PTM, Prosocial Tendencies Measure; DUREL, The Duke University Religion Index; C-PG means communist-prime group; B-PG means Buddhist-prime group; Neu-PG means neutral-prime group. [a] The p-value was obtained using chi-square test; P_1 means the p-value of Study 1; P_2 means the p-value of Study 2.

Supplementary Table 2 The descriptive results of demographic questions and control variables of two studies.

	**Study 3** (n = 311)		P_3	**Study 4** (n = 313)		P_4
	C-PG (n = 155)	Neu-PG (n = 156)		C-PG (n = 154)	Neu-PG (n = 146)	
Sex (male/female)	80/75	73/83	0.40[a]	43/111	52/94	0.15[a]
Volunteering (yes/no)	140/15	144/12	0.53[a]	144/10	135/11	0.72[a]
Political identity (communist/nonparty)	140/15	139/17	0.72[a]	142/12	141/6	0.10[a]
CBS	98.02±13.48	98.83±10.34	0.55	98.57±10.83	98.99±8.63	0.71
PTM	82.51±11.95	81.56±11.36	0.48	81.47±9.59	81.87±12.34	0.76
DUREL	7.16±2.52	7.67±2.50	0.07	7.64±2.90	7.40±2.66	0.46

Note. CBS, Communist Belief Scale; PTM, Prosocial Tendencies Measure; DUREL, The Duke University Religion Index; C-PG means communist-prime group; Neu-PG means neutral-prime group. [a] The p-value was obtained using chi-square test; P_3 means the p-value of Study 3; P_4 means the p-value of Study 4.

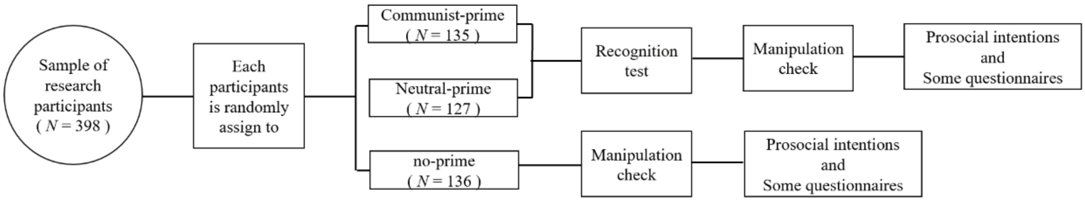

Supplementary Fig. 1. Schematic representation of the experimental procedures of Study 1.

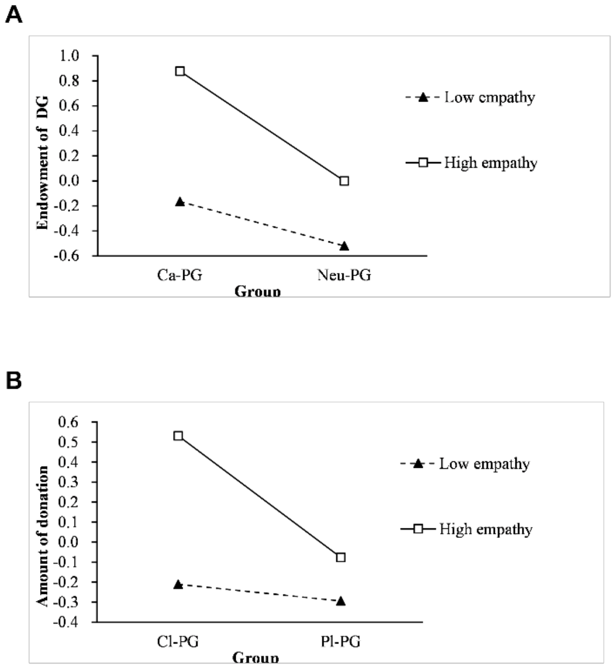

Supplementary Fig. 2. Association for groups (communist-authority-prime, neutral-prime group) and the endowment of DG (A) in Study 3 and Association for groups (communist-authority-prime, neutral-prime group) and the amount of donation (B) in Study 4 at one standard deviation below the mean (low empathy), and one standard deviation above the mean (high empathy) Cl-PG means communist-lecture-prime group; Pl-PG means physics-lecture-prime group.

Supplementary Materials

STUDY 1

1. Video Recall Test

The following questionnaire needed to be completed with your help after you watched the video. Please judge whether the video just played mentioned the following words. If so, please mark the "√" after the words. If not, do not make any mark. Your reply counts for much to us. Please answer following the actual condition.

Personal information:

Age：__________ Gender:________ Major：_________

Class：___________ Politics identity：____________

Have you ever participated in volunteer activities? ___________________________

Words Recall Test:

民族_____ **文化_____** **发展_____**

nation_____ **culture_____** **development_____**

社会主义_____	路径_____	新时代_____
**socialism_____**	**way_____**	**new age_____**
定律_____	折射_____	改革开放_____
**law_____**	**refraction_____**	**reform and opening up_____**
围篱_____	间距_____	计算_____
**fence_____**	**spacing_____**	**calculate_____**
竹子_____	系统_____	传播方向_____
**bamboo_____**	**system_____**	**propagation direction_____**
设置_____	碎片_____	介质_____
**setting_____**	**fragment_____**	**medium_____**
全部清空_____	手锯_____	花园_____
**empty_____**	**hand saw_____**	**garden_____**

2. Manipulation Check

I believe in the principles of the Communist Party.

☐1-----☐2-----☐3-----☐4-----☐5-----☐6-----☐7-----☐8-----☐9

1 representing strongly disagree 9 representing strongly agree

3. Hypothetical Scenarios

Adapted from: Jordan, J., Mullen, E., & Murnighan, J. K. (2011). Striving for the moral self: The effects of recalling past moral actions on future moral behavior. *Personality and Social Psychology Bulletin*, *37*(5), 701-713.

https://doi.org/10.1177/0146167211400208
Nelson, L. D., & Norton, M. I. (2005). From student to superhero: Situational primes shape future helping. *Journal of Experimental Social Psychology*, *41*(4), 423-430. https://doi.org/10.1016/j.jesp.2004.08.003

Here are a few hypothetical scenarios. Please put yourself in your shoes and think about what would you think and do. Give your true answer. Circle the options that best suit your own situation (1 representing very slightly or not at all，5 representing moderate level, 9 representing extremely).

a、The activity is jointly held by the school and welfare home. It needs volunteers to prepare and assist the activity for two hours every two weeks, and the welfare home is eight kilometers away from the school. To what extent would you like to volunteer for the rest of the semester?

□1-----□2-----□3-----□4-----□5-----□6-----□7-----□8-----□9

1 representing very slightly or not at all 5 representing moderate level 9 representing extremely

b、After class, the school calls on everyone to volunteer to stay for one hour to help the school do a thorough clean, and sort out the books in the library. To what extent would you like to volunteer for the rest of the semester?

□1-----□2-----□3-----□4-----□5-----□6-----□7-----□8-----□9

1 representing very slightly or not at all 5 representing moderate level 9 representing extremely

c、During the summer vacation, the school organizes a six-day activity to help the left-behind children in the poor mountainous areas. But you are planning to take a part-time job on vacation. To what extent would you quit your part-time job to participate in poverty alleviation activities?

□1-----□2-----□3-----□4-----□5-----□6-----□7-----□8-----□9

1 representing very slightly or not at all 5 representing moderate level 9 representing extremely

d、Due to the shortage of blood type, the Chine blood bank appeals to the outside world for blood donation. How much would you like to donate 300ml blood on the premise of blood type matching?

□1-----□2-----□3-----□4-----□5-----□6-----□7-----□8-----□9

1 representing very slightly or not at all 5 representing moderate level 9 representing extremely

4. Attitude Survey

Here are some sentences that express some points of view. Please read them carefully and judge whether you agree with these views. Then make a choice at the bottom of the sentence. (1 representing disagree，3 representing neutral, 5 representing agree).

Communist Belief Scale

Taken from: McFarland, S. (1998). Communism as religion. *The international journal for the psychology of religion, 8*(1), 33-48. https://doi.org/10.1207/s15327582ijpr0801_5

题号	题 项（items）	强烈反对	稍微反对	中立	稍微同意	非常同意
1	I believe in the principles of the Communist Party.	1	2	3	4	5
2	I believe in the principles of Marxism-Leninism.	1	2	3	4	5
3	Human society will eventually develop into a communist society	1	2	3	4	5
4	The principles worked out by Marx and Lenin show us the way to solve today's problems.	1	2	3	4	5
5	The main reason for the poor situation of our economy is that it is based on communist principles.	1	2	3	4	5
6	As Lenin said, the capitalist system is the enemy of mankind because it creates antagonisms.	1	2	3	4	5
7	We should continue firmly to follow the communist road of development.	1	2	3	4	5
8	The works of Marx and Lenin may have been progressive for their own day, but for today they are outdated.	1	2	3	4	5
9	The main aim of my life is to build the foundations of communism.	1	2	3	4	5
10	We all must struggle diligently to build a communist society.	1	2	3	4	5
11	Every communist must contribute to the development of communism in other countries.	1	2	3	4	5
12	I try to keep my communist principles in every part of my life at work, in my free time.	1	2	3	4	5
13	I often think about our important problems in building a communist society.	1	2	3	4	5
14	I am certain that my beliefs in communism make me a better person.	1	2	3	4	5
15	People who hold to communist principles are more moral and honorable than others.	1	2	3	4	5
16	My faith in communist ideals helps me find correct solutions in life.	1	2	3	4	5
17	The main reason I believe in communism is that it helps build our national better.	1	2	3	4	5
题号	题 项	强烈反对	稍微反对	中立	稍微同意	非常同意
18	The main reason I believe in communism is that it helps create our national unity.	1	2	3	4	5
19	The main reason I hold to communism is that others expect it of me.	1	2	3	4	5
20	One of the main reasons why I hold to communism is so that I can enjoy the esteem of other people.	1	2	3	4	5

21	It is possible that I would not follow communism if it did not gain my esteem with other people.	1	2	3	4	5
22	The privileges which one receives as a member of the party are too small.	1	2	3	4	5
23	If membership in the party did not provide privileges, I would be less interested in being a member of the party.	1	2	3	4	5
24	I often think about questions of the correctness of my beliefs in communism.	1	2	3	4	5
25	I think that doubts and uncertainties in the truthfulness of communism are essential.	1	2	3	4	5
26	My attitude toward Marxism-Leninism is the result of long thought and doubt.	1	2	3	4	5
27	In order to find the truth of political issues, it is important to continually ask new questions, and not to just accept everything from the work of classical Marxism-Leninism.	1	2	3	4	5
28	Individuals can never arrive at mature political persuasions without questions and doubt.	1	2	3	4	5

Duke University Religion Index

Taken from: Koenig, H. G., & Arndt, B. (2010). The Duke University Religion Index (DUREL): A Five-Item Measure for Use in Epidemological Studies. *Religions, 1*(1), 78-85. https://doi.org/10.3390/rel1010078

题 项（items）	never	once a year or less	less than once a month	once a week	More than once a week
1. How often do you attend group religious meetings? [i.e, mosques, churches, temples, puja, pilgrimage visit, and so forth]	1	2	3	4	5
2. How often do you spend time in private religious activities? [i.e, prayer, meditation, reading scriptures or Bible, worship, and so forth]	few or never	less than once a week	once a week	once a day	more than once a day
	1	2	3	4	5
3. In your life, do you experience the presence of the Divine? [i.e, Allah, Buddha or God and other apparitions]	definitely not true	trends not to be true	unsure	trends to be true	definitely true
	1	2	3	4	5
4. Are your religious beliefs what really lie behind your whole approach to life?	definitely not true	trends not to be true	unsure	trends to be true	definitely true
	1	2	3	4	5
5. Do you try hard to promote your religious beliefs to people around you?	definitely not true	trends not to be true	unsure	trends to be true	definitely true
	1	2	3	4	5

Prosocial Tendencies Measure

Taken from: Carlo, G., & Randall, B. A. (2002). The Development of a Measure of Prosocial Behaviors for Late Adolescents. *Journal of Youth & Adolescence*, *31*(1), 31-44. https://doi.org/10.1023/A:1014033032440

题号	题 项（items）	强烈反对	稍微反对	中立	稍微同意	非常同意

1	I can help others best when people are watching me.	1	2	3	4	5
2	It is most fulfilling to me when I can comfort someone who is very distressed.	1	2	3	4	5
3	When other people are around, it is easier for me to help needy others.	1	2	3	4	5
4	I think that one of the best things about helping others is that it makes me look good.	1	2	3	4	5
5	I get the most out of helping others when it is done in front of others.	1	2	3	4	5
6	I tend to help people who are in a real crisis or need.	1	2	3	4	5
7	When people ask me to elp them, I don’t hesitate.	1	2	3	4	5
8	I prefer to donate money anonymously.	1	2	3	4	5
9	I tend to help people who hurt themselves badly.	1	2	3	4	5
10	.I believe that donating goods or money works best when it is tax-deductible.	1	2	3	4	5
11	I tend to help needy others most when they do not know who helped them.	1	2	3	4	5
12	I tend to help others particularly when they are emotionally distressed.	1	2	3	4	5
13	When I was in the spotlight, it is the time for me to do my best to help others.	1	2	3	4	5
14	It is easy for me to help others when they are in a dire situation.	1	2	3	4	5
15	Most of the time, I help others when they do not know who helped them.	1	2	3	4	5
16	I believe I should receive more recognition for the time and energy I spend on charity work.	1	2	3	4	5
17	Emotional situations make me want to help needy others.	1	2	3	4	5
18	I never hesitate to help others when they ask for it.	1	2	3	4	5
19	I think helping others without giving away my information is the best situation for helping others.	1	2	3	4	5
20	One of the best things about doing charity work is that it looks good on my resume.	1	2	3	4	5
21	I think that helping others without them knowing is the best type of situation.	1	2	3	4	5
22	I often make anonymous donations because they make me feel good.	1	2	3	4	5
23	I feel that if I help someone, they should help me in the future.	1	2	3	4	5

STUDY 2

1. **Lexical Decision Task**

Adapted from: Wittenbrink, B., Judd, C. M., & Park, B. (1997). Evidence for racial prejudice at the implicit level and its relationship with questionnaire measures. Journal of Personality and Social Psychology, 72(2), 262. https://doi.org/10.1037/0022-3514.72.2.262

Communist-prime words:

红军 党徽 党旗 长征 党校 井冈山 中国梦 党中央 解放军 马克思 改革开放 共产党员 从严治党 社会主义 依法治国 为民服务 无产阶级 人民当家 共同富裕 革命先烈

Buddhist-prime words
涅槃 因果 如来 佛陀 皈依 菩提心 如来藏 弥勒佛 金刚经 唯识宗 玄奘法师 达摩祖师 金刚力士 菩萨佛祖 观音菩萨 文殊菩萨 供奉戒律 福慧双修 藏传佛教 大乘佛法

After completing all of the above tests, please answer the following questions:
(1) Do you think there are any rules or themes in sentences during the task?

(2) Do you think the tasks are related to each other

Supplemental File D

STUDY 3

1. Comprehensive Abilities Survey

This research is to investigate the comprehensive abilities of contemporary college students. This study includes three parts, the first part was to investigate language competence, the second part was to measure the economic decision-making ability, and the last part was to assess the attitudes and views of subjects. Please answer the following questions according to your true opinion.

The Scrambled Sentence Task
Adapted from: Srull, T. K., & Wyer, R. S. (1979). The role of category accessibility in the interpretation of information about persons: Some determinants and implications. *Journal of Personality and Social Psychology*, *37*(10), 1660. https://doi.org/10.1037/0022-3514.37.10.1660

Language Proficiency— Communist Prime Group

Here are 20 sets of words that are out of order. You need to construct a coherent and grammatically correct five-word sentence by eliminating one of the words. Write down the number of the correct sentences in the blanks. Note: there is an interference word in each group. Remove the interference word to form a grammatically correct sentence.

1. ① 清新 ② 非常 ③ 公园 ④ 空气 ⑤ 栏杆 ⑥ 雨后的
   ① fresh ② very ③ park ④ air ⑤ ~~handrail~~ ⑥ after the rain
2. ① 常用的 ② 是 ③ 玩具 ④工具书 ⑤ 生活中 ⑥ 汉字词典
   ① common ② is ③ ~~toy~~ ④ Toolbook ⑤ in daily life ⑥ dictionary
3. ① 历史悠久 ② 要 ③ 光荣传统 ④ 我们 ⑤ 红军的 ⑥ 发扬
   ① ~~historic~~ ② should ③ tradition ④ we ⑤ the Red Army ⑥ work on
4. ① 聚焦点 ② 根据地 ③ 红色 ④ 革命 ⑤ 是 ⑥ 井冈山
   ① ~~focus~~ ② foothold ③ red ④ revolutionary ⑤ is a ⑥ Jing Gangshan
5. ① 以便捷 ② 交通工具 ③ 教室 ④ 著称 ⑤ 飞机在 ⑥中
   ① its convenience ② transportations ③ ~~classroom~~ ④ is known for ⑤ airplane ⑥ among
6. ① 特性 ② 耐旱 ③ 仙人掌 ④ 性格 ⑤ 具有 ⑥ 的
   ① tolerant ② drought ③ cactus ④ has ⑤ the features of ⑥ ~~character~~
7. ① 而 ② 为实现 ③ 积极心态 ④ 努力奋斗 ⑤ 中国梦 ⑥ 我们

① to ② are working hard ③ Chinese dream ④ realize ⑤ ~~positive mental attitude~~ ⑥ we

8. ① 磁性最强 ② 叫做 ③ 颜色 ④ 的部分 ⑤ 磁铁上 ⑥ 磁极
① the most magnetic ② is ③ ~~color~~ ④ part ⑤ a magnet ⑥ the pole

9. ① 常见的 ② 说明文 ③ 图书 ④ 一种 ⑤ 文体 ⑥ 是
① ~~book~~ ② expository ③ is ④ a ⑤ common ⑥ style of writing

10. ① 新中国 ② 革命先烈 ③ 或者 ④ 的诞生 ⑤ 换来了 ⑥ 用生命
① ~~or~~ ② the revolutionary martyrs ③ paid with ④ their lives ⑤ for ⑥ the birth of new China

11. ① 思想教育 ② 传统 ③ 重任 ④ 肩负着 ⑤ 党校 ⑥ 的
① ideological education ② ~~tradition~~ ③ the heavy responsibility of ④ shoulders ⑤ Party school ⑥ the birth of new China

12. ① 快于 ② 声音 ③ 方向 ④ 传播速度 ⑤ 的 ⑥ 光
① faster ② than ③ sound ④ ~~direction~~ ⑤ travels ⑥ light

13. ① 伟大 ② 取得 ③ 中国的 ④ 胜利 ⑤ 改革开放 ⑥ 历史
① a great ② has scored ③ China's ④ victory ⑤ reform and opening up ⑥ ~~history~~

14. ① 精神 ② 的 ③ 解放军 ④ 小溪 ⑤ 我们学习 ⑥ 值得
① the spirit ② of ③ PLA ④ ~~stream~~ ⑤ learning ⑥ is worth

15. ① 繁星般 ② 笔记本 ③ 沙滩上 ④ 贝壳 ⑤ 洒满 ⑥ 的
① stars ② ~~notebook~~ ③ the beach ④ seashells ⑤ is strewn with ⑥ like

16. ① 历史上的 ② 人类 ③ 神奇 ④ 是 ⑤ 奇迹 ⑥ 长征
① history ② in human ③ ~~supernatural~~ ④ is ⑤ a miracle ⑥ the Long March

17. ① 哲学思想 ② 创立 ③ 津津乐道 ④ 的 ⑤ 马克思 ⑥ 广为人知
① the philosophy ② founded ③ ~~fascinating~~ ④ that ⑤ Marx ⑥ is well known

18. ① 太阳 ② 云层 ③ 照耀 ④ 穿破 ⑤ 大地 ⑥ 发芽
① the sun ② the clouds ③ shone ④ through ⑤ over the earth ⑥ ~~sprout~~

19. ① 黄色 ② 秋季 ③ 狐狸 ④ 会变成 ⑤ 在 ⑥ 银杏树
①yellow ② autumn ③ ~~foxes~~ ④ turn ⑤ in ⑥ Ginkgo trees

20. ① 是 ② 我 ③ 团体的 ④ 共产党员 ⑤ 优秀的 ⑥ 一名
① am ② I ③ ~~group~~ ④ Communist Party ⑤ excellent member of ⑥ an

Language Proficiency—Neutral-prime Group

1. ① 是 ② 氮气 ③ 打扫 ④ 成分 ⑤ 空气 ⑥ 主要
① is ② nitrogen ③ ~~cleaning~~ ④ constituent ⑤ of air ⑥ the main

2. ① 一座 ② 是 ③ 鲜花 ④ 广州 ⑤ 城市 ⑥ 富有活力的
① a ② is ③ ~~flower~~ ④ Guangzhou ⑤ city ⑥ vibrant

6. ① 的 ② 含有 ③ 维生素 ④ 孕育 ⑤ 猕猴桃 ⑥ 丰富
①is ② in ③ vitamins ④ ~~breeds~~ ⑤ kiwi fruit ⑥ rich

7. ① 伪装技能 ② 具有 ③ 树木 ④ 很多 ⑤ 的 ⑥ 昆虫
①camouflage ② have ③ ~~trees~~ ④ many ⑤ skills ⑥ insects

8. ① 向上爬 ② 一只 ③ 台阶 ④ 蜗牛 ⑤ 的 ⑥ 沿着
①up ② a ③ the steps ④ snail ⑤ ~~sky~~ ⑥ climbs

9. ① 磁性最强 ② 叫做 ③ 颜色 ④ 的部分 ⑤ 磁铁上 ⑥ 磁极
①the most magnetic ② is ③ ~~color~~ ④ part ⑤ a magnet ⑥ the pole

10. ① 各式各样 ② 收藏了 ③ 书籍 ④ 的 ⑤ 作画 ⑥ 图书馆
①a great variety ② has ③ books ④ of ⑤ ~~painting~~ ⑥ the library

11. ① 安静 ② 的 ③ 极了 ④ 像 ⑤ 夜晚 ⑥ 校园
①quiet ② is ③ very ④ ~~like~~ ⑤ the night ⑥ on campus

12. ① 是 ② 动物 ③ 冰块 ④ 卵生 ⑤ 企鹅 ⑥ 一种

①is ② animal ③ ~~ice~~ ④ egg-laying ⑤ penguin ⑥ an

13. ① 垃圾 ② 属于 ③ 电池 ④ 回收 ⑤ 输入 ⑥ 不可

①waste ② are ③ batteries ④ recycling ⑤~~input~~⑥ not

14. ① 左右 ② 平均寿命 ③ 了 ④ 两百年 ⑤ 在 ⑥ 胡杨树

①about ② the average life span of ③ ~~boats~~ ④ 200 years ⑤ is ⑥ a poplar tree

15. ① 黄色 ② 秋季 ③ 狐狸 ④ 会变成 ⑤ 在 ⑥ 银杏树

①yellow ② autumn ③ ~~foxes~~ ④ turn ⑤ in ⑥ Ginkgo trees

16. ① 太阳 ② 云层 ③ 照耀 ④ 穿破 ⑤ 大地 ⑥ 发芽

① the sun ② the clouds ③ shone ④ through ⑤ over the earth ⑥ ~~sprout~~

14. ① 以便捷 ② 交通工具 ③ 教室 ④ 著称 ⑤ 飞机在 ⑥中

① its convenience ② transportations ③ ~~classroom~~ ④ is known for ⑤ airplane ⑥ among

15. ① 特性 ② 耐旱 ③ 仙人掌 ④ 性格 ⑤ 具有 ⑥ 的

② tolerant ② drought ③ cactus ④ has ⑤ the features of ⑥ ~~character~~

16. ① 快于 ② 声音 ③ 方向 ④ 传播速度 ⑤ 的 ⑥ 光

① faster ② than ③ sound ④ ~~direction~~ ⑤ travels ⑥ light

17. ① 繁星般 ② 笔记本 ③ 沙滩上 ④ 贝壳 ⑤ 洒满 ⑥ 的

① stars ② ~~notebook~~ ③ the beach ④ seashells ⑤ is strewn with ⑥ like

18. ① 常见的 ② 说明文 ③ 图书 ④ 一种 ⑤ 文体 ⑥ 是

① ~~book~~ ② expository ③ is ④ a ⑤ common ⑥ style of writing

19. ① 清新 ② 非常 ③ 公园 ④ 空气 ⑤ 栏杆 ⑥ 雨后的

a) fresh ② very ③ park ④ air ⑤ ~~handrail~~ ⑥ after the rain

20. ① 特性 ② 耐旱 ③ 仙人掌 ④ 性格 ⑤ 具有 ⑥ 的

~~①~~ tolerant ② drought ③ cactus ④ has ⑤ the features of ⑥ ~~character~~

2. Economic Decision-making Ability

Please read the rules of the game carefully. You will receive their show-up fee of CNY20. After the game, each student will get a cash reward according to the total number of tokens they got in the game (1 token = 0.2CNY).

The Dictator Game

Adapted from: Kahneman, D., Knetsch, J. L., & Thaler, R. H. (1986). Fairness and the assumptions of economics. *Journal of business*, S285-S300.

Now we are playing a game, there will be two roles in the game process, A and B. A must decide how to distribute a sum of money between herself or himself and a second player, B. B must accept the A's decision. A and B in the study were randomly selected and paid according to the decision made by A.

This time you are A, and you have been given 100 tokens. You can send some, all, or nothing to another person that you have randomly been paired with. The other person has not received any money. The money you will have after your decision is 100 tokens minus the amount you send to the other person. The other person will only have the amount you send. We assure you that the results of the assignment will be anonymous.

How many tokens do you decide to contribute to B?____________(0-100)

Interpersonal Reactivity Index

Taken from: Davis, M. H. (1980). A Multidimensional Approach to Individual Differences in Empathy. *Journal of Personality & Social Psychology*, *10*(85).

题号	题 项（items）	强烈反对	稍微反对	中立	稍微同意	非常同意
1	I daydream and fantasize, with some regularity, about things that might happen to me.	1	2	3	4	5
2	I often have tender, concerned feelings for people less fortunate than me.	1	2	3	4	5
3	I sometimes find it difficult to see things from the "other guy's" point of view.	1	2	3	4	5
4	Sometimes I don't feel very sorry for other people when they are having problems.	1	2	3	4	5
5	I really get involved with the feelings of the characters in a novel.	1	2	3	4	5
6	In emergency situations, I feel apprehensive and ill at ease.	1	2	3	4	5
7	I am usually objective when I watch a movie or play, and I don't often get completely caught up in it.	1	2	3	4	5
8	I try to look at everybody's side of a disagreement before I make a decision.	1	2	3	4	5
9	When I see someone being taken advantage of, I feel kind of protective towards them.	1	2	3	4	5
10	I sometimes try to understand my friends better by imagining how things look from their perspective.	1	2	3	4	5
11	I sometimes try to understand my friends better by imagining how things look from their perspective.	1	2	3	4	5
12	Becoming extremely involved in a good book or movie is somewhat rare for me.	1	2	3	4	5
13	When I see someone get hurt, I tend to remain calm.	1	2	3	4	5
14	Other people's misfortunes do not usually disturb me a great deal.	1	2	3	4	5
15	If I'm sure I'm right about something, I don't waste much time listening to other people's arguments.	1	2	3	4	5
16	After seeing a play or movie, I have felt as though I were one of the characters.	1	2	3	4	5
17	Being in a tense emotional situation scares me.	1	2	3	4	5
18	When I see someone being treated unfairly, I sometimes don't feel very much pity for them.	1	2	3	4	5
19	I am usually pretty effective in dealing with emergencies.	1	2	3	4	5
20	I am often quite touched by things that I see happen.	1	2	3	4	5
21	I believe that there are two sides to every question and try to look at them both.	1	2	3	4	5
22	I would describe myself as a pretty soft-hearted person.	1	2	3	4	5
23	When I watch a good movie, I can very easily put myself in the place of a leading character.	1	2	3	4	5
24	I tend to lose control during emergencies.	1	2	3	4	5
25	When I'm upset at someone, I usually try to "put myself in his shoes" for a while	1	2	3	4	5
26	When I am reading an interesting story or novel, I imagine how I would feel	1	2	3	4	5

	if the events in the story were happening to me.					
27	When I see someone who badly needs help in an emergency, I go to pieces.	1	2	3	4	5
28	Before criticizing somebody, I try to imagine how I would feel if I were in their place	1	2	3	4	5

After completing all of the above tests, please answer the following questions:

(1) Do you think there are any rules or themes in sentences during the language task?

(2) Do you think the tasks are related to each other

STUDY 4

1. Charitable Appeal

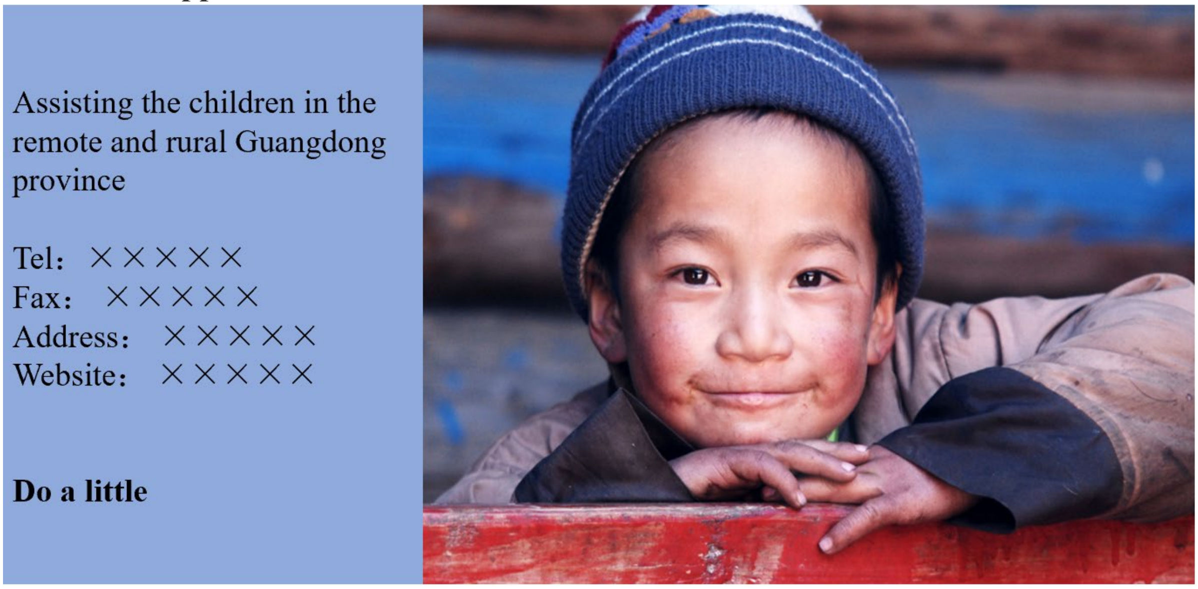

Reproduced with permission from Baidu Picture.
